# Supplementary material for: Association of macular choroidal thickness with optical coherent tomography morphology in patients with idiopathic epiretinal membrane
Source: PLoS One. 2020 Sep 29;15(9):e0239992. doi: 10.1371/journal.pone.0239992 (PMC7523999; doi:10.1371/journal.pone.0239992)
Supplement: Attachment — Submitted filename: respond to reviewer.docx [file pone.0239992.s001.docx]

July 5, 2020

Dear editor:

Thank you and the reviewers for the comprehensive review and valuable comments on our manuscript entitled “Association of Macular Choroidal Thickness with Optical Coherent Tomography Morphology in Patients with Idiopathic Epiretinal Membrane ‘’. We have revised the paper in accordance with the editor’s suggestions. In the following pages we will address each comment one by one. We appreciate the comments from the reviewers to improve the quality of our article. We hope our paper will be acceptable to be published in “*PLOS ONE* ’’.

Respectfully yours,

I-Mo Fang, M.D, Ph.D.

TEL: +886-2-27861288 ext.8275

FAX: +886-2-27888492

E-mail: dah75@tpech.gov.tw

**Reviewer 1:**

Reviewer’s opinion 1#:

Anatomical speaking the fovea is 1.5mm in diameter while foveal avascular zone is only 0.5mm. Therefore, the notion that fovea receives 100% of its blood supply from choroidal is not correct.

Responses:

We totally agree and thanks to reviewer’s comment. The foveola is approximately 0.35 mm in diameter and lies in the center of the [fovea](https://en.wikipedia.org/wiki/Fovea_centralis) and contains only [cone cells](https://en.wikipedia.org/wiki/Cone_cell). .In this region the cone receptors are found to be longer, slimmer and more densely packed than anywhere else in the retina, thus allowing that region to have the potential to have the highest visual acuity in the eye. We have changed the wording “ fovea” into “ foveola” in line 1, page 5.

Reviewer’s opinion 2#:

The hypothesis that the fovea is vascularized by choroid therefore the epiretinal membrane should have choroidal changes is counterintuitive. The vice versa would be more relevant.

Responses:

We very much agree with reviewer. Several studies have reported that the tangential and vertical traction of ERM may cause retinal folds, macular edema, and vascular distortion, leading to altered hemodynamics in macular areas. Membrane contraction of ERM patients contributes to the increase in choroidal thickness in idiopathic ERM patients.

Reviewer’s opinion 3#:

There are multiple reports on choroidal changes in ERM and novelty of the work is minimal. PMID: 31671195, PMID: 30467423, PMID: 26338821, PMID: 31139433, PMID: 28245442.

Responses:

Actually, the changes of choroidal thickness or circulation is a hot toptic. Although there are various articles published on this topic, the focus of these articles is different. The title of PMID: 31671195 is “Macular Choroidal Thickness Changes in Development, Progression, and Spontaneous Resolution of Epiretinal Membrane”. This paper mainly focus on evaluating the association between macular choroidal thickness and the development, progression, and resolution of epiretinal membrane (ERM). The authors found that ERM with thining choroid is easy to progress, and ERM with thick choroid is easy to spontaneous resolve. The title of PMID: 30467423 is “Macular Microvasculature Features Before and After Vitrectomy in Idiopathic Macular Epiretinal Membrane: An OCT Angiography Analysis”. This study was to evaluate pre-operative and post-operative vascular parameters changes in ERM by optical coherence tomography angiography (OCTA). The title of PMID: 26338821 is Choroidal Thickness Change Following Vitrectomy in Idiopathic Epiretinal Membrane and Macular Hole. In this study, patient with macular hole were included, but our study were not. The title of PMID: 31139433 is “Mapping Diurnal Variations in Choroidal Sublayer Perfusion in Patients With Idiopathic Epiretinal Membrane: An Optical Coherence Tomography Angiography Study”. This study investigated diurnal changes in choroidal sublayer perfusion in eyes with ERM. In our study, we divided the patients into three morphological groups according to OCT findings. : Group 1, ERM without membrane contraction; Group 2, ERM with membrane contraction and retinal folding; and Group 3, ERM with membrane contraction and macular edema. The above studies did not have the same research as ours. Therefore, I believe that our research conclusion was still quite clinically valuable. Thanks reviewer for your reminder.

Reviewer’s opinion 4 #:

Only figure 1A was showing the EDI images and the rest are very poor quality.
Responses:

We thanks reviewer for the comment. Because the output graphic of OCT is a PDF file, the patient has cataract... and other factors, resulting in poor resolution of the images. We have try to revise the figure.

Reviewer’s opinion 5 #:

Figures:

1. Figure 1. Poor scan quality with no visible choroidal structure in B and C, questioning the EDI mode of the scan.

2. Figure 3. Again, very poor contrast of the images makes it hard to evaluate choroidal thickness analysis.

Responses:

We have try to revise the figure 1 and 3.

**Reviewer 2:**

Thanks reviewer for your encouragement.
